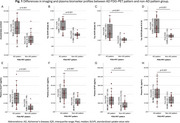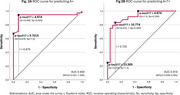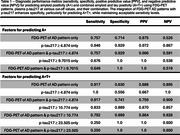# Alzheimer's and Non‐Alzheimer's FDG‐PET Patterns: Biomarker Differences and Diagnostic Performance for Amyloid and Tau Pathology

**DOI:** 10.1002/alz70856_098861

**Published:** 2025-12-24

**Authors:** Kittithatch Booncharoen, Poosanu Thanapornsangsuth, Watayuth Luechaipanit, Thanaporn Haethaisong, Adipa Chongsuksantikul, Prawit Oangkhana, Yuttachai Likitjaroen, Akarin Hiransuthikul, Sekh Thanprasertsuk

**Affiliations:** ^1^ Neurology Center, Phyathai 1 Hospital, Bangkok, Rachathewi, Thailand; ^2^ Neurocognitive Unit, Division of Neurology, Department of Medicine, Faculty of Medicine, Chulalongkorn University, Bangkok, Thailand; ^3^ Memory Clinic, Division of Neurology, King Chulalongkorn Memorial Hospital, Bangkok, Thailand; ^4^ Thai Red Cross Emerging Infectious Diseases Health Science Centre, World Health Organization Collaborating Centre for Research and Training on Viral Zoonoses, King Chulalongkorn Memorial Hospital, The Thai Red Cross Society, Bangkok, Thailand; ^5^ Memory Clinic, King Chulalongkorn Memorial Hospital, The Thai Red Cross Society, Bangkok, Thailand; ^6^ Division of Neurology, Department of Medicine, Faculty of Medicine, Chulalongkorn University, Bangkok, Thailand; ^7^ Thai Red Cross Emerging Infectious Diseases Health Science Centre, King Chulalongkorn Memorial Hospital, Bangkok, Thailand; ^8^ Thai Red Cross Emerging Infectious Diseases Health Science Centre, King Chulalongkorn Memorial Hospital, The Thai Red Cross Society, Bangkok, Thailand; ^9^ Department of Preventive and Social Medicine, Faculty of Medicine, Chulalongkorn University, Bangkok, Thailand; ^10^ Department of Physiology, Faculty of Medicine, Chulalongkorn University, Bangkok, Thailand

## Abstract

**Background:**

FDG‐PET has been a widely used imaging modality for evaluating hypometabolism in Alzheimer's disease (AD) for decades. This study investigates differences in biomarker profiles between patients with AD and non‐AD patterns of FDG‐PET. Additionally, we aim to explore whether the AD FDG‐PET pattern complements the diagnostic performance of plasma *p*‐tau217 for detecting beta‐amyloid (Aβ) and tau pathology.

**Method:**

We included 51 participants diagnosed with amnestic mild cognitive impairment or mild dementia. The participants were classified by radiologist‐reported FDG‐PET patterns as either AD (*n* = 32) or non‐AD (*n* = 19). AD patterns were defined by typical AD‐related hypometabolism (involving posterior cingulate, precuneus, and posterior temporal and parietal lobes). Aβ‐PET centiloid (AβCL), Aβ positivity (A+), tau standardized uptake value ratio (SUVR) in Braak III and/or IV, tau‐PET status (T+), and plasma biomarkers, including *p*‐tau217, *p*‐tau181, glial fibrillary acidic protein (GFAP), and neurofilament light chain (NfL) levels, were determined. ROC analyses identified optimal cut‐off values for *p*‐tau217, which were further assessed alongside FDG‐PET patterns for their diagnostic performance for A+ and A+T+.

**Result:**

Participants with AD FDG‐PET patterns, compared to those with non‐AD patterns, exhibited higher proportions of A+ (28/32 (87.5%) vs. 9/19 (47.4%), *p* = 0.002) and A+T+ (22/32 (68.8%) vs. 2/19 (10.5%), *p* <0.001). They also showed elevated AβCL levels and tau SUVRs across Braak III‐IV regions (all *p* <0.001, Figure 1A‐1D). Plasma biomarkers, including *p*‐tau217, were markedly higher in the AD pattern group (all *p* <0.001, Figure 1E‐1H). The AD FDG‐PET pattern demonstrated high diagnostic accuracy, while plasma *p*‐tau217 alone (at optimal cut‐off values) showed excellent performance for predicting A+ and A+T+ (Table 1). Combining FDG‐PET patterns with *p*‐tau217 (cut‐off ≥ 4.874 pg/mL) enhanced specificity for A+T+ prediction (from 0.556 to 0.741) while maintaining high sensitivity (from 1.0 to 0.917) (Table 1). The determination of *p*‐tau217 cut‐off values will be illustrated with ROC curves (Figure 2A‐B).

**Conclusion:**

FDG‐PET patterns, particularly when combined with plasma *p*‐tau217, improve diagnostic specificity for A+ and A+T+ pathology. These findings emphasize the complementary value of integrating imaging and fluid biomarkers for accurate AD diagnosis.